# Clinical implementation of the smartphone app Robin Z as an additional treatment tool to support adolescents with psychiatric symptoms

**DOI:** 10.1192/j.eurpsy.2023.1819

**Published:** 2023-07-19

**Authors:** N. Traber-Walker, E. Voets, C. Bühlmann, M. Gerstenberg, F. Probst, M. Franscini, S. Walitza

**Affiliations:** Department of Child and Adolescent Psychiatry and Psychotherapy, University of Zurich, Zurich, Switzerland

## Abstract

**Introduction:**

Interest in the development of innovative technologies in the health sector has increased due to their potential to improve accessibility, efficacy, quality, and cost-effectiveness of treatment. Based on these considerations, we developed the app Robin Z to support adolescents in psychiatric treatment. Robin Z is intended as an add on therapy-tool. It aims to assess symptoms in real time, offer help in coping with symptoms and everyday life and to support medication adherence. Despite initial encouraging research findings supporting the use of smartphone technology in psychotherapy, it remains unclear whether the consistent use of smartphone technology in outpatient clinics is practical outside of research projects. Thus, it is uncertain whether patients will engage with this technology over an extended period of time and whether clinicians will be willing to integrate this new technology into their routine. In view of these factors, it is crucial to evaluate the use of smartphone apps for their applicability, effectiveness, and efficiency in clinical routine. In our investigation, we want to address these questions and fill the gap between research and clinical practice.

**Objectives:**

The aim of our evaluation is to identify barriers in clinical implementation plus to assess the usability and applicability of the Robin Z app in clinical practice.

**Methods:**

We started the clinical implementation of Robin Z in four community-based outpatient services. We collected data of 27 adolescent patients and their caregivers (N=15) over a six-week period. They all completed questionnaires on user-friendliness and satisfaction. Further, user data about mood logs, symptom trajectories, achieved weekly goals and entries for positive reinforcement were gathered to examine the clinical impact of using the app.

**Results:**

The clinical implementation and evaluation will provide data on feasibility, user-friendliness, clinical implication and satisfaction of patients and therapists with the smartphone app Robin Z.

**Conclusions:**

Although many apps are available for young people with mental health problems, most of these have not been developed by professionals, and their effectiveness has not been evaluated. To the best of our knowledge, Robin Z is one of the first apps of its kind to be specifically developed by clinical experts as an additional tool to support psychotherapy for adolescent patients. The results of this evaluation are of clinical importance to the field of eMental Health. They will provide preliminary evidence of the clinical utility of the app. In addition, the results will improve our understanding of potential barriers and facilitators to using Robin Z for both patients and therapists.

**Disclosure of Interest:**

None Declared

